# Preparation and Applications of Electrospun Optically Transparent Fibrous Membrane

**DOI:** 10.3390/polym13040506

**Published:** 2021-02-08

**Authors:** Yanan Xiao, Hao Luo, Rongxing Tang, Jiazi Hou

**Affiliations:** Key Laboratory of Automobile Materials, Ministry of Education, College of Materials Science and Engineering, Jilin University, Changchun 130025, China; ynxiao20@mails.jlu.edu.cn (Y.X.); haoluo@jlu.edu.cn (H.L.); tangrx20@mails.jlu.edu.cn (R.T.)

**Keywords:** electrospinning, optically transparent, nanofibrous membrane

## Abstract

The optically transparent electrospun fibrous membrane has been widely used in many fields due to its simple operation, flexible design, controllable structure, high specific surface area, high porosity, and unique excellent optical properties. This paper comprehensively summarizes the preparation methods and applications of an electrospun optically transparent fibrous membrane in view of the selection of raw materials and structure modulation during preparation. We start by the factors that affect transmittance among different materials and explain the light transmission mechanism of the fibrous membrane. This paper also provides an overview of the methods to fabricate a transparent nanofibrous membrane based on the electrospinning technology including direct electrospinning, solution treatment after electrospinning, heat treatment after electrospinning, and surface modification after electrospinning. It further summarizes the differences in the processes and mechanisms between different transparent fibrous membranes prepared by different methods. Additionally, we study the utilization of transparent as-spun membranes as flexible functional materials, namely alcohol dipstick, air purification, self-cleaning materials, biomedicine, sensors, energy and optoelectronics, oil–water separation, food packaging, anti-icing coating, and anti-corrosion materials. It demonstrates the high transparency of the nanofibers’ effects on the applications as well as upgrades the product performance.

## 1. Introduction

Electrospinning, referred to electrostatic spinning, is one of the simple and effective methods to prepare continuous fibers with diameters ranging from nanometers to micrometers. Different from the traditional spinning method, electrospinning is a process in which polymer solution or melt is stretched by means of high-voltage electric field and finally solidified into fibers [1]. Electrospun fibrous membrane materials have been widely used in biomedicine, photoelectric materials, filtration, and adsorption due to their flexible design, controllable structure, high specific surface area, good adsorption, and permeability [2,3,4].

**Figure 1:**
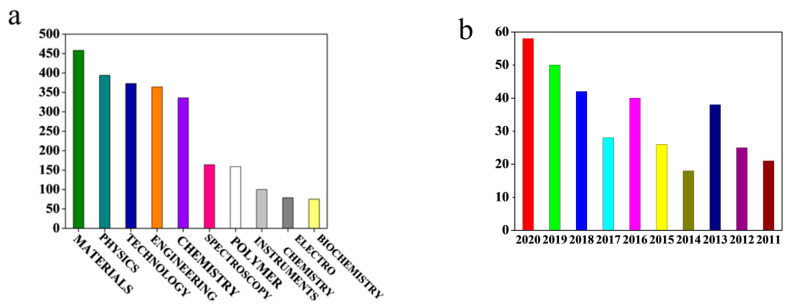
(**a**) Quantitative statistics of related articles on transparent as-spun nanofibers literature in various academic fields. (**b**) Statistics on the number of articles on electrospun transparent fiber membranes published each year in the last 20 years.

In particular, transparent as-spun membranes have attracted public attention due to their excellent optical properties. It is worth noting that in 1999, the first article on a transparent electrospun membrane was published, in which Bergshoef and Vancso impregnated the as-spun PA-4,6 nanofibers with resin, drying, and curing for 60 h, and they obtained the transparent fiber membrane [5]. In the ensuing 20-year period, the number of published articles about transparent electrospun membranes has been increased from 1 to 55 a year, which implies that the preparation and application of transparent electrospun fibrous membranes have attracted the attention of researchers. Among them, research studies on transparent fiber materials are mainly concentrated on materials science, accounting for 74.164% of the total literature (as shown in Figure 1).

During the last decade, the transparency of the as-spun membrane was based on transparent raw materials and structure modulation. As a transparent material, polystyrene (PS) nanofiber prepared by electrospinning was a transparent matrix to encapsulate CsPbBr_3_ nanocrystals (NCs). These made a type I core–shell structure. [6]. In order to modulate the structure easily, researchers were more likely to choose easy processing and soluble materials, such as polyethylene terephthalate (PET). Du [7] used the spectrophotometer to test the light transmittance of the material and found that the microstructure such as the areal density of PET nanofibers was significantly changed by adjusting the concentration of the PET spinning solution. As the areal density increased, the transmittance decreased. The main reason is that with the areal density increasing, the refractive indices of air and fibers at the interface increase. When electrospinning TiO_2_, Li [8] observed that with the electrospinning time extension, the thickness of the membrane increased and the transmittance gradually decreased. Hence, many factors determine the transmittance of an as-spun membrane, such as the refractive indices, the thickness of the membrane, and the transparency of raw materials. Moreover, there are common methods to test the light transmittance of an as-spun membrane, namely spectrophotometer [9], transmittance meter [10], and integrating sphere photometer [11]. The above methods are noninvasive testing technologies, and the membrane structure is not destroyed. All in all, appropriate material selection, structure modulation, and testing methods contribute to the development of preparation and characterization of transparent as-spun membranes. 

**Figure 2:**
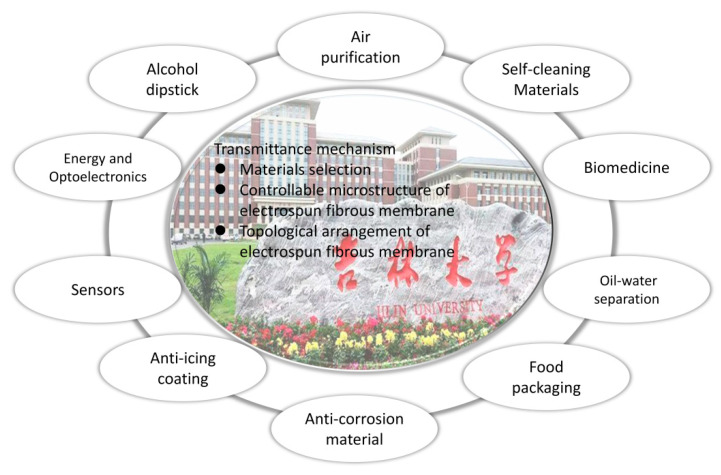
The applications of transparent as-spun membranes.

Apart from this, the increase demand on various fields has fueled the development of the transparent fibrous materials. On the one hand, the improvement of light transmittance broadens applications of fibrous membrane, such as transparent wound dressing, which not only promotes wound healing but also provides a rapid visual window for wound detecting, so as to avoid the secondary injury of patients caused by its frequent opening–closing [12]. On the other hand, the application of transparent fibrous membranes improves the performance of products. As far as optical fibrous solar cells are concerned, excellent optical performance can effectively improve the energy utilization and photoelectric conversion efficiency [13]. In addition, according to the change of transparency, it directly observes membrane durability and confirms the product life for the timely replacement of the material, such as filtration and adsorption materials [14,15]. Therefore, in the light of the above advantages, the development of electrospun fibrous membrane with excellent light transmission performance will broaden the application scope of micro-/nano- membrane materials and promote the technological upgrading (as shown in Figure 2).

In recent years, some researchers studied electrospun nanofibrous membranes with high transmittance used for various applications involved in air purification, biomedicine, sensors, etc. However, there is no review to summarize light transmission mechanism, raw materials, fabrication techniques, and applications. This article could benefit beginners who are eager to become familiar with electrospun optically transparent fibrous membranes, having preliminary understanding and moving to a deeper mastery of that.

## 2. Optically Transparent Materials

Transmittance refers to the percentage of the transmitted light flux to the incident luminous flux on the surface of transparent materials [16]. It is an important performance to characterize the degree of transparency of materials. The higher the transmittance, the more transparent the materials. For various materials, such as metals, ceramics, and polymers, according to the difference of bonds, microstructure, and properties, the factors that affect transmittance are different, varying from material to material. 

### 2.1. Ceramic Materials

Most ceramic materials are opaque in the visible light range. Only by adjusting the crystal structure by physical or chemical means can the materials show high transmittance. According to Rayleigh scattering theory, the size of nanocrystals must be far smaller than the wavelength of visible light, and the refractive index of crystal phase must be close to that of the matrix [17,18,19] in order to prepare transparent crystal materials. In other words, the optical transparency of ceramics is closely related to the size and concentration of nanocrystals.

The transparency of ceramic materials largely depends on the optical anisotropy of residual pores and defects, second phase inclusions, rough surfaces, and crystals [20,21,22,23]. As a result, by controlling the content of additives, the transmittance of ceramic materials can also be very high. Miao et al. [24] explored the relationship between the addition of calcium fluoride (CaF_2_) and light transmittance by changing the amount of CaF_2_ in transparent glass–ceramic (TGCs) materials. When the CaF_2_ content was 15.4 wt %, 20.4 wt %, and 35.4 wt %, the aluminum silicate polycrystalline crystals were formed in the glass ceramic sample, and the TGCs were opaque. When the CaF_2_ content increased to 27.4 wt %, CaF_2_ was used as a nucleating agent to make the glass matrix crystallize early. Then, nanocrystals were formed through heat treatment with the size much smaller than the wavelength of visible light. Meanwhile, the refractive index of the crystal phase was close to that of the glass matrix, which was in accordance with Rayleigh scattering theory. Therefore, the TGCs were highly transparent.

The reason for the low light transmittance of dielectric ceramics is the various light-scattering locations, such as residual pores on rough surfaces and second phase at grain boundaries [25,26,27]. Zhang et al. [28] improved the compactness of the (K_0.5_Na_0.5_) NbO_3_ (KNN) matrix and reduced the porosity of the crystal by reducing the grain size and removing the impurities in the crystal. The transmittance of the KNN-based ceramics had been significantly improved. Belyaev et al. [29] prepared Co: ZnAl_2_O_4_ transparent ceramics by the hot-pressing method and explored the influence of the content of cobalt (Co) and sintering additive (ZnF_2_) on the ceramics transparency. It was found that when the content of Co and ZnF_2_ was in the range of 3–10 wt %, the average grain size was in the range of 50–70 mm, which made ceramics compact on the micro-scale and highly transparent on the macro-scale. Chen et al. [30] synthesized nanopowders by the co-precipitation method and then fabricated Ce: Gd_2_YGa_3_Al_2_O_12_ transparent ceramics by the two-step-sintering that is pre-sintering and hot-isostatic-pressing. The key to achieving transparency is high density, fine grains, and little residual intergranular pores, which can be tailored by controlling the sintering process. 

In conclusion, it was indispensable to maximize the size of nanocrystallines smaller than wavelengths of visible light, lower the porosity, and increase the density. 

### 2.2. Metal Materials

With a special structure, a fraction of metal materials and organometallic compound is transparent, such as nanoparticles [31], nanonets [32], and nanowires [33,34]. Most incident light can pass through the metal grids to achieve high transmittance, while only a little incident light is refracted and lost. Kubwimana [35] prepared a metal conductive grid on the top of the Lexan substrate with a transmittance of 84.5%. Owing to the fact that transparency is the percentage ratio of no metal areas to metal areas, the array antenna reached high transmittance by adjusting the pitch of mesh material. Therefore, the structure of metal materials has an important impact on the transmittance. Lee et al. [36] prepared Ag nanowires (AgNWs) transparent films with excellent photoelectric properties and introduced the value of merit (*FoM*) to define the trade-off between conductivity and optical transparency.

(1) $$FoM=\frac{188.5}{\mathrm{Rs}\left(T^{-\frac{1}{2}}-1\right)}$$

where Rs is the sheet resistance and T (at λ = 550 nm) is transmittance. The formula quantitatively displayed that the larger the *FoM* value, the lower the sheet resistance and the higher the transmittance.

Oxygen doping is also an effective method to reduce metal optical loss and improve transmittance. Zirconium (Zr) is an opaque metal in the visible light range. Under the two-step oxidation process combining air heat treatment and low oxygen partial pressure, Zr metal foil can be prepared into doped and transparent monoclinic ZrO_2_, and the transmittance reached 60%. The monoclinic transparent ZrO_2_ was composed of equiaxed grains of tens of nanometers and columnar crystals of several microns in length [37]. Additionally, Jo et al. [38] used oxygen as dopant in the silver metal layer. The average transmittance of the optimized silver metal layer was 93.5% in the range of 500–800 nm, and the optical loss was reduced to 1.01%.

Improving the dispersion of metal on the carrier is also an effective way to improve the transmittance of organometallic compound. Wu et al. [39] used the impregnation deposition method to load Pt catalytic centers on Mo_2_C. There was a strong electronic effect between Mo_2_C and Pt clusters, and Pt clusters had small size, good dispersion, and low loading on the carrier, which prevented Pt aggregation from absorbing and scattering light. 

### 2.3. Polymer Materials

Different from metal and ceramic materials, the transparency of polymer materials mainly depends on its crystallinity [40]. The polymers with high crystallinity will increase light reflection, resulting in high light loss and low light transmittance. Polycarbonate (PC), an amorphous polymer with low crystallinity, has high light transmittance and could be used as a transparent matrix. For instance, Park et al. [41] adopted a biomass-derived isosorbide instead of bisphenol A to prepare a PC nanocomposite with higher transmittance than pure PC. Moreover, PET [42], PMMA [43], and PS [44] are also introduced as a transparent matrix for their low crystallinity. Kim et al. [45] mimicked the surface structure of the Progomphus obscurus (sanddragon) wing, physically killing the attached bacteria, and they prepared a transparent, colorless, and self-disinfecting polyethylene terephthalate (PET) film by plasma treatment. 

**Figure 3:**
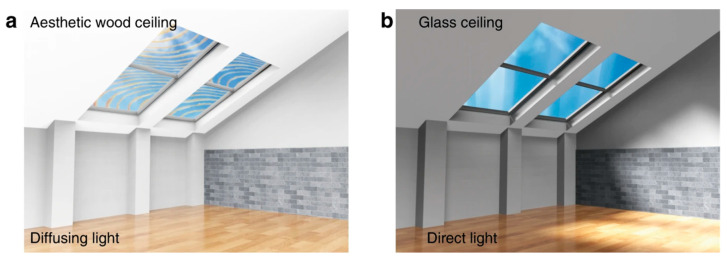
The schematic scene shows the light distribution and aesthetic appeal inside a building via applying (**a**) the aesthetic wood ceiling comparing with (**b**) the glass ceiling (Adapted from [50]).

For other opaque polymers, chemical treatment and composite modification are needed to obtain transparent products. For example, the optical transparence of natural wood could be significantly improved by the chemical removal of chromogenic groups and lignin, and the penetration of polymers with a suitable refractive index, such as polyvinyl alcohol (PVA) [46,47], epoxy resin [48] and polyimide (PI) [49]. Using epoxy resin as a permeation polymer, Mi et al. [50] prepared transparent wood with light transmittance up to 80%, which has a good anti-glare performance and light-guiding properties (as shown in Figure 3). By the means of chemical treatment, the light transmittance of PI could be very high in the visible light range. Polyimide (PI) usually appears dark brown because of the charge transfer complex formed between the highly conjugated substituents imino group and the rigid benzene ring, which will introduce a monomer with a strong electron withdrawing group in the main chain of PI, preventing the electron movement on the main chain of PI [51,52]. Therefore, optically transparent PI (CPI) can be synthesized by changing the monomer structure. Kim and Chang [53] blended poly (amic acid) (PAA) and PVA with PI, and then, they heat-treated them to obtain a blend film of CPI and PVA. After removing PVA, a porous CPI film with high transmittance was obtained.

Inorganic nanofillers with a high refractive index also effectively improve the transmittance of polymers. Tao et al. [54] filled the high refractive index TiO_2_ filler into the poly (glycidyl methacrylate) (PGMA) polymer chain through covalent action, which realized the space-shielding effect, reduced light scattering, and prevented the aggregation of inorganic nanoparticles. The light transmittance of the prepared polymer material was as high as 90%.

## 3. Light Transmission Mechanism of Electrospinning Fibers

Due to the flexible selection of an electrospun matrix, it is easily to obtain various fibrous membranes made of ceramics, metals, and polymers. However, the enhanced transparency of an electrospun fibrous membrane is still a challenge because of its unique interconnected networks. The main reason is that when the incident light hits an interface, the light will spread in different forms, including transmission, reflection, refraction, scattering, or absorption. Among them, the reflection of light is the main factor causing light loss, and with the increase of the interface area, the light loss continues to increase [55]. 

Electrospinning membranes have numerous interconnected pores, which inclines to form dozens of air–fiber interfaces. Therefore, a large amount of reflection/refraction loss occurred at these interfaces, which eventually made the membrane opaque. So, the effective method to reduce the light loss is adjusting the pores of electrospun fibrous membrane and building an interface with little light loss. The reflection of incident light at the interface is related to the refractive index of the two heterogeneous materials forming the interface [56], which is expressed by the following formula:

(2) $$\Gamma ={\left[\frac{n_{1}-n_{2}}{n_{1}+n_{2}}\right]}^{2}$$

where Γ is the reflection coefficient, and *n*_1_ and *n*_2_ are the refractive indices of air and fibers at the interface, respectively. Since the refractive index difference between the air and fibers leads to the increase of the reflection, reducing the refractive index difference of the heterogeneous materials at the interface improves the optical transparency of the fibrous film.

There are two kinds of transmittances: specular transmittance and diffusive transmittance. For specular transmittance, the photo-detector only measures the light that transmits along the same axis as the incident. For diffusive transmittance, the transmitted light is collected at all angles by an integrating sphere [57].

When a photon encounters an object, it can interact with it via refection, adsorption, or refraction. However, light scattering, especially forward scattering (namely diffusive transmittance), will cause an optical haze effect that possesses excellent optical management capability including the anti-glare effect and light guiding, which are of great significance for transparent ceiling applications but are detrimental for indoor displays. The light management of transparent substrates is extremely important for optoelectronic devices. For example, an increased absorption path of light in a solar cell will enhance the device’s conversion efficiency; thus, a considerable light scattering is preferred. An anti-glare coating is needed for display-driven devices to function in bright environments, such as for touch screens in global positioning systems (GPS). For such applications, an anti-glare coating with large light scattering is required. Therefore, it is very important to be able to tailor the light scattering of the transparent substrates for different applications.

**Figure 4:**
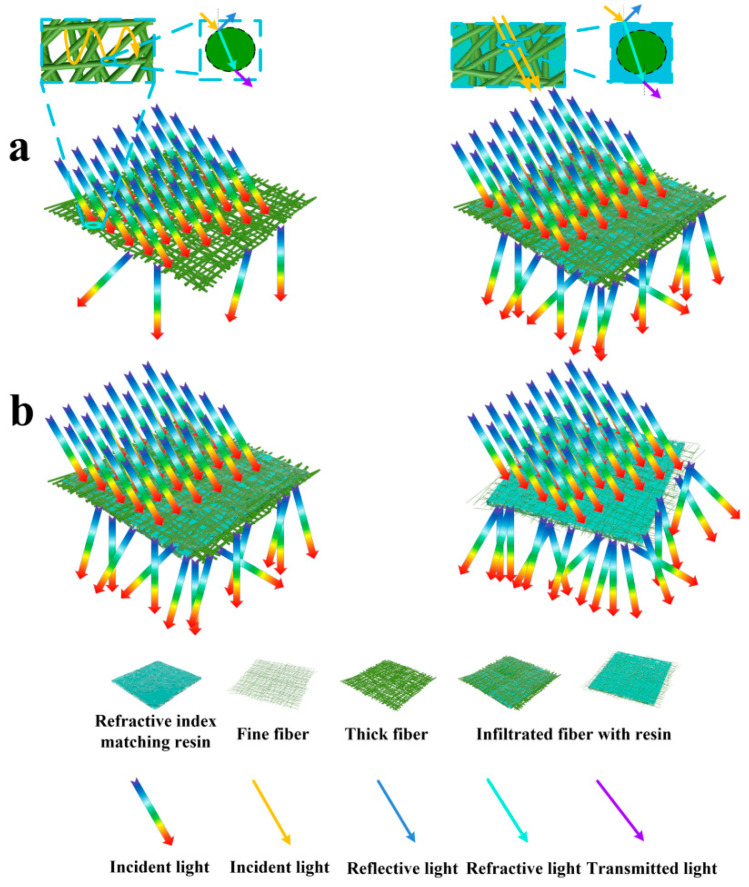
Schematic diagram of the influence of refractive index, (**a**) electrospun fibers diameter and (**b**) refractive index on light propagation (the number of arrows represents light intensity).

Other factors such as the nanofibers’ diameter have also occupied a prominent position because it would have an effect on the propagation process of light. Light is an electromagnetic wave in theory; thus, visible light can pass fibers of <400 nm in diameter without the occurrence of reflection and/or refraction at the fiber/matrix interface. Light undergoes reflection and/or refraction upon reaching nanofibers whose diameters are larger than 400 nm. The total diffusive transmittance does not vary much as the fiber diameter is smaller than 400 nm. However, specular transmittance and the haze values change dramatically in the visible wavelength range. Li et al. [58] utilize a self-blending co-electrospinning (SBCE) to prepare aligned fiber-hybrid mats consisted of “uniaxially” oriented and well-mingled PA-6 nanofibers as the reinforcement and PMMA microfibers as the matrix. The PA-6/PMMA fiber-hybrids are subsequently fabricated into optically transparent nanocomposite sheets by hot press molding. Thereafter, the nanofiber directionality effects on the optical transmittance and mechanical properties of the thus-produced PA-6/PMMA nanocomposite in two orthogonal directions are sequentially evaluated. They find that transmittance of the 1% PA-6/PMMA nanocomposite can be remarkably enhanced with PA-6 nanofibers prepared at increasing rotating speeds. This is because higher rotating speeds facilitate the generation of nanofibers with a decreased diameter (as shown in Figure 4).

Therefore, according to the light transmission mechanism of electrospinning fibers, some research studies on material selection and structure modulation are conducted to obtain an as-spun fibrous membrane with high transparency.

### 3.1. Materials Selection

Many common materials including polycarbonate (PC), polyethylene terephthalate (PET), polymethyl methacrylate (PMMA), polystyrene (PS), and other materials such as nylon, epoxy resin, and thermoplastic polyurethane are optically transparent. Using transparent raw materials could obtain electrospun fibrous membrane with high light transmittance directly. For example, the raw material of thermoplastic polyurethane (TPU) fibrous membrane is transparent with a light transmittance of 90%, which still maintains a high light transmittance after electrospinning [10]. PS is one of the five transparent plastics with a light transmittance of more than 90%. The modified PS fibrous film via electrospinning also showed excellent optical transparency [59].

However, the transparency of most electrospun fibrous membranes is not high. To improve the light transmittance of electrospun fibers, it can be achieved by compounding with transparent polymer materials. The common methods include impregnation, hot pressing, surface modification, etc. For instance, a polyacrylonitrile (PAN) electrospun fibrous membrane is white in the visible light range, while it shows excellent optical transparency after being immersed in PMMA solution with high transmittance. The transmittance of the composite membrane in the visible light range increased to 90% [55] (as shown in Table 1). The transparency and mechanical properties of PA-6/PMMA transparent composite fibrous membrane with a core–shell structure are much higher than that of the PA-6 nanofibrous membrane because of the more uniform distribution and less aggregation of PA-6 nanofibers in the core–shell structured fibers membrane [60].

### 3.2. Controllable Microstructure of Electrospun Fibrous Membrane

**Figure 5:**
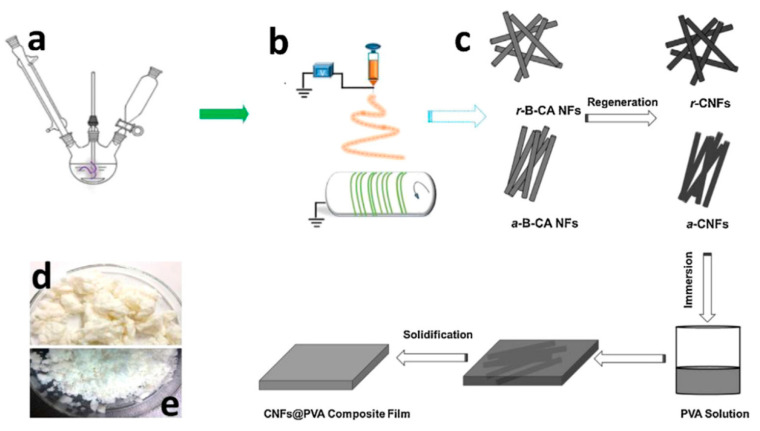
Schematic illustration of (**a**) acetylation reaction, (**b**) electrospinning, (**c**) CNFs@PVA composite films preparation process, and (**d**,**e**) photographs of bamboo cellulose (BC) and B-CA (Adapted from [61]). CA: cellulose acetate, PVA: polyvinyl alcohol (Adapted from [61]).

Packing density or excellent interfacial adherence among fibers could not be ignored for light transmittance, because the air within the nanofibers internal structure will increase light scattering and reduce the transmittance. Cai et al. [61] deacetylated bamboo source cellulose to prepare cellulose acetate (CA) material. By adjusting the receiving device, the nanofibers with uniaxial arrangement were fabricated. Then, the fibrous membrane was immersed in PVA solution. Since the refractive index difference between CA fibers and PVA resin is far less than that between CA and air, a composite membrane with a light transmittance of 75% was obtained (as shown in Figure 5). 

### 3.3. Topological Arrangement of Electrospun Fibrous Membrane

For the material without transparency, the design of its topological arrangement can reduce the membrane thickness and effectively improve its transmittance. Polycaprolactone (PCL) is a kind of degradable plastic, and it can be electrospun into white fibers with low transmittance [62]. Pan et al. [63], inspired by the woven structure of the window screen, improved the preparation method of a PCL electrospun fibrous membrane, and electrospun it to deposit fibers with anisotropic thickness gradients. Most of the fibers were densely packed on the wires in a small area, while very few fibers were sparsely suspended in the voids over a large area. (Most of the fibers were densely distributed on the wire mesh, and a few of them were sparsely suspended in the space among the wire meshes.) Light was easy to pass through the suspended fibers, which greatly improved the overall light transmittance of the macro structure of the fibrous membrane, compared with the isotropic fibrous membrane, showing optical transparency.

## 4. Preparation Methods of Transparent Electrospun Fibrous Membrane

Several research studies are developed to achieve transparent electrospun fibrous membranes. Direct electrospinning and post treatment after electrospinning as the main methods are employed to increase the optical transparency of the fibrous membrane.

### 4.1. Direct Electrospinning

Direct electrospinning heavily depends on the selection of high light transmittance materials, while the fiber diameter and film thickness also have a significant impact on the light transmittance [64]. Li et al. [8] prepared a kind of self-cleaning transparent titanium dioxide (TiO_2_) nanofilm on the glass substrate by direct electrospinning. The diameter of the fibers was 50 nm, and the thickness of the film was 5 μm. The specific method was to add a series of different contents of diethanolamine (DEA) into the polymer polyvinylpyrrolidone (PVP) solution of TiO_2_, place the transparent glass substrate in front of the aluminum foil to collect, and finally remove organic components to get TiO_2_ nanofilm by high-temperature sintering. With the content of DEA increasing, the morphology of the films deposited on the slide changed from opaque to transparent. Additionally, adjusting the electrospinning time to control the film thickness is also an effective method to improve the transmittance. Chen et al. [10] prepared an elastic TPU electrospun fibrous membrane with a diameter distribution of 0.82 ± 0.22 μm; by collecting for 10 min, the fibrous membrane was transparent in the visible light range, and the light transmittance reached more than 60%.

In addition, polymethyl methacrylate (PMMA) and its modified materials are often used as the matrix of direct electrospinning to prepare high-transmittance fibrous films. PMMA is transparent because of its large side group, irregular crystal structure, and complete light transmission. For the electrospun PMMA, its fiber diameter is smaller than the wavelength of visible light, and the transmission light loss is less, so it has a slight influence on the light transmittance [65]. Examples are the AgNWs [66], n-butyl acrylate/methyl methacrylate copolymer (BA-co-MMA) electrospun fibrous membrane [67], polymethylmethacrylate/tungsten disulfide (PMMA/WS_2_) composite nanofibrous membrane [68], and tungsten oxide/organic framework/polymethylmethacrylate (WO_2_/PEDOT/PMMA) conductive nanohybrid fibers [69]. 

Furthermore, Gan and Zhang [70] fabricated thermoplastic polyurethane (TPU) nanofiber/net membranes with high transparency by electrospinning. Adopted DMF and acetone as solvent, TPU elastomer, and various LiCl salt amounts were put into the mix to prepare the precursor. Ruan et al. [71] obtained the transparent PAN: TiO_2_ and polyacrylonitrile-co-polyacrylate: TiO_2_ (PAN-co-PMA: TiO_2_) nanofiber membranes composite nanofiber membranes by direct electrospinning as well. TiO_2_, PAN, PAN-co-PMA, and PVP were added to the DMF solution to prepare the mixed procedure. The obtained membranes exhibited high transparency and excellent air permeability. Meanwhile, Salehi [72] adopted poly-ε-caprolactone (PCL)–silkfibroin (SF) as raw materials to fabricate a hybrid scaffold through electrospinning. In comparison with non-aligned scaffolds, the aligned PCL-SF scaffolds displayed higher transparency, hydrophilicity, and water uptake.

Other transparent electrospun systems, such as nano diamond/nylon-11/polyacrylonitrile (ND/PA-11/PAN) [73], keratin/polyvinyl alcohol/carbon quantum-dot (keratin/PVA/c-dot) [74], can also be directly electrospun into transparent fibrous membranes. The transmittance of all above materials is up to 82%.

### 4.2. Solution Treatment after Electrospinning

#### 4.2.1. Solution Casting

**Figure 6:**
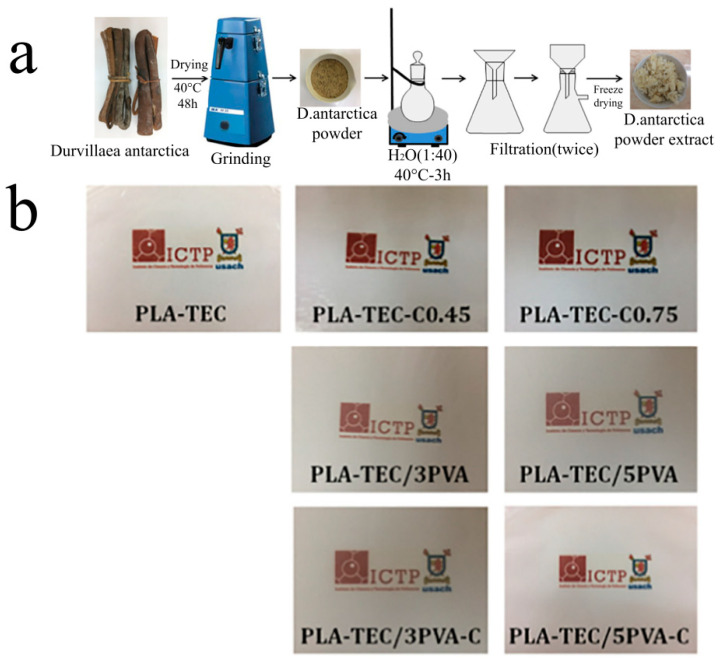
(**a**) Schematic representation of extraction procedure of *D. Antarctica* algae from natural algae to powder extract and (**b**) visual appearance of the obtained bionanocomposite films. (adapted from [75]).

Solution casting is the process of dispersing the electrospun fibers in the matrix solution with high transmittance by stirring or ultrasonic techniques. With the casting solution evaporating and drying, the transparent membrane is obtained. This method is usually used for the preparation of electrospun reinforcement materials. Jiang et al. [64] cut nylon-6 (PA-6) nanofibers by electrospinning to obtain discontinuous short fibers with an average diameter of 163 ± 4.73 nm and length ranging from tens of microns to hundreds of microns and dispersed them in TPU and PMMA solutions, respectively. After casting and drying, PA-6/TPU and PA-6/PMMA nanofibers film were obtained simultaneously. The test results showed that PA-6 short fibers had no significant effect on the transparency of TPU and PMMA films compared with the electrospun continuous fibers, and the light transmittance of the composite film was still over 86%. Arrieta et al. [75] added D. antarctica, an extract from Antarctic alga, into PVA solution, obtaining D. antarctica/PVA composite fibers. Subsequently, it was dispersed in polylactic acid and formed a flexible and optically transparent fibrous membrane through casting (as shown in Figure 6). 

#### 4.2.2. Dipping

The non-transparent electrospun fibrous membrane can optimize its optically transparence by solution dipping. The method is to soak the as-spun fibrous membrane in a transparent solution and then dry the membrane after the fibrous membrane is fully soaked in the solution. The transparent solution fills the pores among the fibers, which greatly improves the light transmittance of the composite membrane.

The PVA solution has good transparence after drying, so it is often used as an impregnation solution of electrospun membrane. Najarzadekan and Sereshti [76] immersed the opaque polycaprolactam/1, 10-phenanthroline (PA-6/1, 10-phen) hybrid electrospun fibers into PVA aqueous solution, evaporated the solvent, and obtained a highly transparent fibrous membrane. Additionally, Stachewicz et al. [77] also used PVA as the impregnation solution and electrospun nylon-6 (PA-6) fibers as the reinforcement fibers to prepare optical transparent film by dipping. Moreover, the effect of PVA concentration in the impregnation solution on the light transmittance of the fibrous membrane was also discussed. The increase of PVA concentration led to more PVA entering into the porous nanofibers, the porosity of the film decreased, and the optical transparency increased. When the concentration of PVA reached 20 wt %, PVA filled all the pore networks, and the light transmittance was the highest. When that of PVA was greater than 20 wt %, the excess PVA adhered to the surface, increasing the membrane thickness, and the transparency of the composite membrane began to decline.

**Figure 7:**
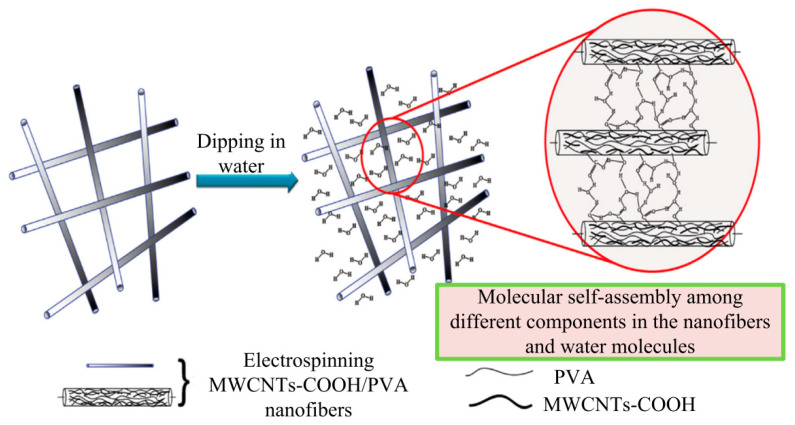
Scheme of self-assembly of transparent conductive composite films from nanofibers (Adapted from [79]).

Other impregnation solutions, such as epoxy resin or PMMA, can also improve the light transmittance of the electrospun fibrous membrane. Liao et al. [78] used thermoset epoxy resin and curing agent as the impregnation matrix. Uniaxially oriented as-spun cellulose nanofibers were soaked in the above mixed solution and solidified at a certain temperature. After the treatment, the membrane with a light transmittance of 88–92% was developed. Wu et al. [55] compounded an opaque PAN fibrous membrane and PMMA impregnated solution by solution dipping. The optical transparency of the PAN fibrous membrane was improved, and the light transmittance was up to 90% in the visible light range. Carboxylated multi-wall carbon nanotubes (MWCNTs-COOH)/PVA nanofibers prepared by direct electrospinning exhibited hydrophilicity and swelling properties. Therefore, the structure of the fibrous membrane was changed due to the aqueous solution dipping and nanofibers swelling to obtain a transparent membrane with a dense pore-free structure (as shown in Figure 7). Its transmittance reached 71% [79].

In addition, the heated solvent vapor as an alternative dipping candidate, the transparent fibrous membrane can be obtained by the solvent evaporation treatment. Ma et al. [80] prepared a sandwich structured fibrous membrane composed of PCL and shellac. It has good mechanical properties and light transmittance after being treated with ethanol steam, which can be used for drug release in skin care.

### 4.3. Heat Treatment after Electrospinning

#### 4.3.1. Hot Pressing after Electrospinning

Under certain temperature and pressure conditions, an electrospun fibrous membrane and other matrix materials are pressed to form composite membrane. The as-spun fiber is distributed in other materials as a dispersion phase. After removing pressure, a highly transparent composite fibrous membrane is obtained. Hot pressing can promote the material to fill the gap between the fibers. It can also reduce the light loss in the pores and therefore improve the light transmittance. Moreover, hot pressing between the two fibrous membranes but not the fibrous membrane/matrix materials can also be conducted. Kurokawa and Hotta [81] used electrospinning to prepare stereocomplex polylactide (sc-PLA) reinforced poly(l-lactide) (PLLA), and they obtained the self-reinforced PLA with a light transmittance of more than 75% by hot pressing. PLLA was infiltrated into an sc-PLA nanofibers membrane after melting at high temperature, filling the gap between sc-PLA fibers and improving the light transmittance.

In order to avoid the decrease of light transmittance caused by crystallization, the fibrous film was quenched in liquid nitrogen immediately after compression molding to reduce the effect of secondary crystallization on the light transmittance. Lu et al. [82] prepared the thermochromic hydrophobic transparent nanocomposites by electrospinning and hot pressing, using PMMA and VO_2_ nanoparticles as raw materials. Li et al. [83] prepared a Cr/PA-6/PMMA ternary system electrospun fibrous membrane with significantly improved tensile strength and fracture toughness by hot pressing. During the preparation process, PA-6 was dissolved in Cr/hexafluoroisopropanol (HFIP) solution and then mixed with PMMA for electrospinning. PMMA fibers were fused to form a continuous matrix phase, and Cr/PA-6 as the reinforcement phase improved the mechanical properties of the composite fibrous. Compared with the unreinforced pure PMMA, the transparency loss of the composite nanofibrous membrane was less than 10%, and the transmittance of the membrane was still above 70%. An et al. [84] prepared different proportion nylon-6,6 and cyclobutylene terephthalate (CBT) composite membranes by hot pressing. The results showed that the transmittance of the nanocomposite film with 76% CBT was the highest, because CBT, as a continuous matrix phase, filled the gap between nanofibers, resulting in a sharp reduction in the surface roughness of the composite fibrous film, avoiding light scattering and thus improving the transparency of the composite [74]. Due to temperature and pressure, hot pressing sometimes makes the material melting and cross-linking, forming a dense fibrous membrane.

Jiang et al. [85] immersed the electrospun PA-6 nanofibrous membrane in different concentrations of melamine formaldehyde (MF) aqueous solution and then prepared the fibrous membrane material by hot pressing. The PA-6/MF composite fibrous membrane was constructed by polymerization and cross-linking of MF around nylon-6 nanofibers under hot pressing. The effect of the increase of PA-6 fibers content on the transmissivity of the composite was also investigated. The higher the fibers content, the lower the transmissivity of the fibrous membrane.

#### 4.3.2. Annealing after Electrospinning

Annealing is a process of heat treatment in which the material is exposed to high temperature for a period of time and then cooled slowly. As a common means of heat treatment, the annealing process is widely used to eliminate the defects inside the metal [86], so as to improve the performance of the material. This method also plays a certain auxiliary role in improving the optical transparency of polymer materials.

**Figure 8:**
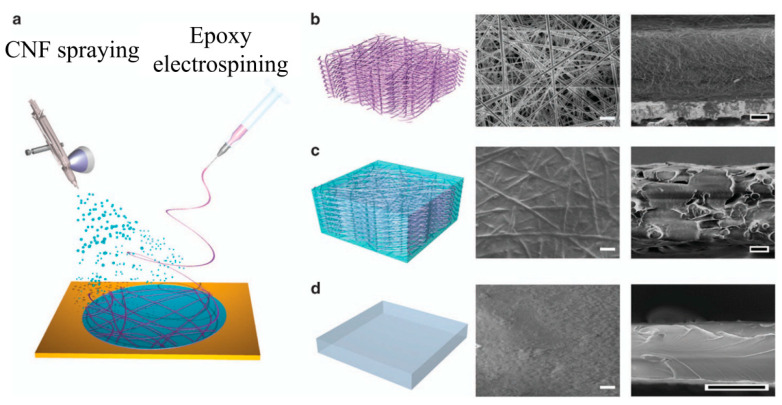
Preparation of the cellulose nanofibers (CNF) hybrid film. (**a**) A schematic image of an electrospinning epoxy backbone and spraying CNF fillers simultaneously. Schematics and scanning electron microscopy (SEM) images (top and cross-sectional views) of (**b**) the electrospun three-dimensional (3D) epoxy nanoweb structure, (**c**) the CNF epoxy hybrid before hot pressing, and (**d**) the CNF–epoxy hybrid after hot pressing. White scale bars are 10 μm, and black scale bars are 20 μm. (Adapted from [92]).

Polyvinylpyrrolidone (PVP), a non-ionic polymer compound, is applied for the electrode materials preparation as an electrospun nanofibers matrix. Yoon and Kim [87] fabricated the PVP/ITO (indium tin oxide) as-spun membrane by annealing. The ITO solution was mixed with the PVP solution and then electrospun to obtain as-spun nanofibers, which were heat-treated by an IR furnace at 450 °C for 2 h. The fibrous film with a light transmittance of more than 90% was obtained. Ning et al. [88] also selected PVP as an as-spun matrix and silver nitrate (AgNO_3_)-doped Zn (NO_3_)_2_ as an additive to fabricate the fibrous membrane with a cross-arrangement pattern. After annealing, the optical transparent Ag-doped ZnO nanofibers were obtained. Lamastra [89] et al. directly electrospun nickel acetate and PVP electrospinning precursor solution on sputtering nickel and nickel oxide film and then obtained the transparent and conductive NiO nanofibrous film by annealing in the air in the temperature range of 400–500 °C. The transmittance of nickel oxide film increased from 54% to 75% by annealing. In addition, Cherpinski et al. [90] electrospun poly (β-hydroxybutyrate) (PHB) nanofibrous film and explored the effect of annealing time and cooling method on the optical properties of PHB fibers. The experimental results showed that the minimum annealing temperature for producing uniform transparent film was 160 °C. Wagner et al. [91] also adopted PHB as raw materials to obtain as-spun PHB-/PVA fibers and then dried them thoroughly and annealed them to obtain an ultra-thin fibrous membrane with a thickness of 173 ± 4 μm. This transparent material had potential applications in the delivery of bioresponsive drugs. Furthermore, Ji [92] made a surface modification on electrospinning epoxy nanofibers. The cellulose nanofibers (CNF) were sprayed on the epoxy nanofibers surface by air gun, and the hybrid film was obtained by annealing at 90 °C. The epoxy resin was polymerized by exposure to ultraviolet radiation. Compared with the original CNF film, the light transmittance of the hybrid film was improved significantly from 77% to 88%, which was due to the fact that compared with the CNF nanofibrous film, there were fewer holes in the hybrid film, resulting in a dense accumulation structure and significantly reduced light scattering (as shown in Figure 8). Meanwhile, compared with the original epoxy resin, the mechanical properties of the CNF hybrid film were also significantly improved due to the formation of high-strength carbon fibers. Shin [93] et al. fabricated moiré-fringeless TCFs with good optoelectrical characteristics and excellent thermal stability using a single-pass printed random serpentine network of medium-field electrospun silver microfibers (AgMFs), which would be used as flexible electronic devices. A transparent graphene skin electrode was also fabricated by annealing after electrospinning [94]. The precursor was prepared by dissolving styrene ethylene butylene styrene (SEBS), CuCl_2_, and phenolic resin (PR) into tetrahydrofuran (THF) solvent. A transparent graphene skin electrode was obtained after annealing for half an hour at 600 °C. Its transparency could reach 83%.

Different from traditional conventional thermal annealing (CTA), microwave annealing was an emerging annealing method. Hong [95] applied microwave annealing to post treatment indium tin oxide (ITO) nanofibers fabricated by electrospinning. The process adopted vacuum rapid thermal annealing (RTA) at 300 °C for 30 s, and it increased the electron concentration and reduced the resistance, thereby improving the properties of ITO nanofibers.

#### 4.3.3. Thermal Treatment after Electrospinning

The optical transparency of electrospun fibers also is improved by thermal treatment, such as heating and calcinations. Different from annealing, this kind of heat treatment takes a very short time and is mostly instantaneous heating. Mele et al. [96] obtained the poly(ethyl 2-cyanoacrylate) continuous fibers by electrospinning. After heat treatment on a hot plate at 150 °C for 20 s, a transparent fibers texture coating was obtained, which has good antifouling function. Hsieh et al. [97] successfully electrospun Ag^+^ containing nanofibers from the solution of polyacrylonitrile (PAN)/dimethylacetamide/tetraaniline (TeAN). After heat treatment at 500 °C for a short period of time, the nanofibers on glass slides were successfully transformed into highly transparent and conductive silver nanofibrous networks. Zhao et al. [98] also used PAN as as-spun raw materials and prepared the composite nanofibrous membrane with a light transmittance of 95% by electrospinning the precursor of PAN/PU and heat treated the composite nanofibrous membrane at 200 °C for 10 min. Moreover, Cho and Kuo [99] prepared ZnO nanofibers doped with Al by electrospinning and heating. By adjusting the doping amount of Al in the fibrous membrane, the influence of Al content on fibrous particle size and optical/electrical properties were studied. The results showed that 2% nanofibers had the best electrical properties, and the light transmittance remained around 84%. 

### 4.4. Electroless Deposition after Electrospinning

Electroless deposition is a method to selectively catalyze the metallization of electrospun nanofibers in which the specific operation is inserting electrosensitizers into polymer electrospinning solutions in order to obtain metal nanofibrous materials [100]. Electroless deposition can achieve uniform coating and retain the large surface area inherent in small diameter fibers, without the need of current, ultra-high vacuum, or high temperature [101,102]. 

Hsu et al. [103] prepared metal nanofibrous network (MNWs) by electrospinning and electroless deposition, which was a transparent electrode material with excellent conductivity. Firstly, the electrospun PVB/SnCl_2_ nanofibers were immersed in silver nitrate solution, and the electrospun fibrous membrane with a silver seed layer on the surface was obtained by chemical reduction. The silver seed layer was mainly to facilitate the subsequent electroless deposition process. The prepared nanofibers had 90% light transmittance and good electrical conductivity. Testa et al. [104] prepared junctionless metal nanowire network in this method of the combination of electrospinning and electroless deposition, and the whole process does not require thermal annealing or a vacuum environment. The light transmittance of preparation of metal nano fibrous network was as high as 90%, and the photoelectric performance would be enough to replace indium tin oxide (ITO) and become transparent electrode materials, whose production cost is much lower than the ITO.

### 4.5. Surface Modification after Electrospinning

After the surface modification of the electrospun fibrous film, the new properties of the material were obtained without affecting the optical transparency, such as sputtering coating [105,106], layer-by-layer self-assembly (LBL) [107], in situ polymerization [75], electron beam deposition [108], spraying, etc. 

Jo et al. [109] sputtered platinum (Pt) seeds onto the surface of PAN nanofibers by sputtering and coating and then adjusted the thickness of polymer nanofibers by copper plating on the surface. The self-melting junction formed by electroplating significantly reduced the contact resistance between the copper fibers. In the process of copper plating, the thickness of the fibrous film was controlled by adjusting the plating time to minimize the loss of light transmittance. The fibrous film had a light transmittance of about 90%. 

Recently, Jiang et al. [110] modified the surface of copper electrospun nanofibers (CuNFs) by the functionalization approach, namely chemical reduction, and obtained an Ag-coated core–shell structure nanofibrous membrane. The fibrous membrane had high transmittance and can be transferred to a flexible substrate to make a transparent conductive electrode (TCEs). The transparent conducting electrode based on a Cu/Ag core–shell nano network had good flexibility, transparency, and conductivity, which could be used in new flexible LED devices and solar cells. Sue et al. [111] also adopted the functionalization approach to prepare carbon nanotube/copper (CNT/Cu) fibers after electrospinning. In order to improve the CNT−Cu interactions, cysteine was grafted to the surface of CNTs, and it exhibited not only excellent optoelectrical properties but was also optically transparent. Beregoi et al. [112] fabricated core–double shell nylon-ZnO/polypyrrole electrospun nanofibers by functionalization after electrospinng as well. Nylon 6/6 nanofibers were obtained through electrospinning and then were functionalized with ZnO by a sol–gel process. Furthermore, the ZnO-coated networks had the deposition of a polypyrrole layer as an electrode to cover the nylon–ZnO nanofibers by electrodeposition. The unique core–shell nanofibers were highly transparent and flexible with a wide range of applications.

An et al. [113] prepared a core–-shell Cu/Ni electrode with high conductivity and corrosion resistance by electrospinning and electroplating. Firstly, PAN nanofibers were prepared by electrospinning, and then Cu and Ni were electroplated on the nanofibrous membrane in turn. Not only did the Cu/Ni nanofibers haved high bending resistance and but also theits light transmittance was as high as 93%, which could be used as a transparent heater. 

## 5. Application of Transparent Electrospun Fibrous Membrane

### 5.1. Alcohol Dipstick

As a fairly new application area, rapid visual alcohol dipstick has just emerged in the transparent as-spun membranes application. Recently, the design of alcohol testing devices has attracted the attention of researchers due to the large number of traffic accidents caused by drunk driving. Our group [114] proposed a simple and straightforward method to fabricate electrospun poly (lactic acid)/polyvinylidene fluoride (PLA/PVDF) membrane and studied its transparency wetted by alcohol, which exhibited superior transparency, exceeding 90%. The high transmittance was realized by the approximation of refractive index between the fibers and alcohol penetrated in the pore of the membrane. In comparison with traditional alcohol testing devices, this PLA/PVDF alcohol dipstick was reusable and more responsive to alcohol.

### 5.2. Air Purification

**Figure 9:**
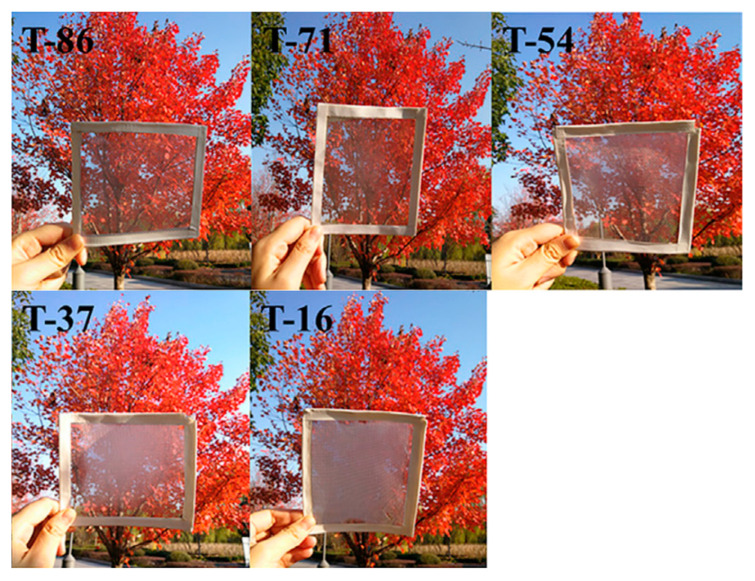
Photographs of PDMS/PMMA–chitosan transparent air filters at different transparencies (adapted from [116]). PMMA: polymethyl methacrylate.

The air purification capacity and efficiency of electrospun fibers can be adjusted by controlling the diameter of the fibers, and the adsorption capacity of polar nanofibers to particulate pollutants is better than that of non-polar nanofibers [14]. As a result of its unique optical property, a transparent fibrous membrane can make light pass through an electrospun membrane effectively, further expanding the application of an electrospun fibrous membrane in PM2.5 adsorption [115]. Recently, Chen et al. [10] studied the influence of the diameter of electrospun TPU fibers on the filtration efficiency. By changing the concentration of TPU in polymer solution, the average diameter of TPU nanofibers increased from 0.14 ± 0.06 μm to 0.82 ± 0.22 μm. The optimized TPU nanofibrous membrane can effectively remove PM_2.5_ from the air. Its efficiency was up to 98.92% and its service life was longer than the commercial filters on sale. Liu et al. [116] developed the PMMA/polydimethylsiloxane (PDMS) super hydrophobic layer and chitosan super hydrophilic layer of double-layer electrospun fibrous membrane materials, with a light transmittance of 86%. Even after 100 h of testing in the air atmosphere with PM_2.5_ > 3000 μgm^−3^, the capture efficiency of the fibrous membrane for PM_2.5_ was still higher than 98.23%, as shown in Figure 9. Liang et al. [117] prepared a transparent TPU air filter with a rotating bead spinel, and the removal efficiency of PM_2.5_ could be as high as 99.654%. The durability of the air filter had also been greatly improved. After 10 times of filtration, it was found that the removal efficiency of PM_2.5_ only decreased by 1.6%. The transparent electrospinning fibrous material also had an important application in the anti-haze window screen. Du et al. [7] attached polyethylene terephthate (PET) to polyester mesh, and the solvent incomplete evaporation could enhance the combination of electrospun polyester nanofibers and polyester mesh, which also increased the number of micron-nanometer holes, improving the light transmittance of window screens; the removal effect of PM_2.5_ was as high as 87%.

An ultra-thin nanofibrous membrane was prepared by electrospinning PVP aqueous solution. In order to prevent the ultra-thin fibrous membrane from being easily damaged by accidental air movement or a stray electric field, Mikheev et al. [118] manufactured the water-soluble nanofilters for collecting biological micro- and nano-aerosols by electrospinning PVP solutions in different solvent mixtures. The electrospun PVP nanofibers were neutralized with a cloud of small counter-ions generated by electrospraying a volatile solvent. The nanoparticle with a diameter of only 0.03 to 0.05 μm can be collected by the fibrous membrane.

### 5.3. Self-Cleaning Materials

The self-cleaning material can keep itself clean on a wide variety of environments, and it has multiple functions such as antifouling, deodorizing, and antibacterial. According to the different wettability of water, self-cleaning materials can be divided into superhydrophobic and superhydrophilic materials. These two types of materials can clean themselves according to the interaction with water [119]. The self-cleaning characteristics of superhydrophobic materials are determined by their high contact angle with water, which makes water form spherical droplets on its surface that are easy to roll away with dust and dirt [120]. On the contrary, on the super hydrophilic surface, water can diffuse completely and form a thin layer. In the presence of suitable photocatalysts, such as sunlight and water, most of the dirt on the surface of the material can be chemically decomposed.

Koo et al. [59] prepared a polystyrene/phenylsilsesquioxane (PS/PhSSQZ) nanofibrous membrane by electrospinning and thermal annealing, which had good environmental stability. The PS/PhSSQZ fibrous membrane had super hydrophobicity and lipophilicity due to its hierarchical fibrous structure and unique chemical properties. It could be used as a self-cleaning material in a hydrophobic structure coating, oil–water separation membrane, etc. After heat treatment at 600 °C, the material still maintained its original fibrous structure, superhydrophobic property, and good stability. Li et al. [8] studied the hydrophilicity and photocatalytic properties of DEA/TiO_2_ films prepared by electrospinning. The photocatalytic properties of this new DEA/TiO_2_ film were studied by Congo red dye decay and silver ion reduction experiments. The experimental results showed that the DEA/TiO_2_ film had an excellent photocatalytic activity. Through the measurement of water contact angle, it was proved that the water contact angle of the super hydrophilic coating was 2 ± 1°, which had a self-cleaning function.

### 5.4. Biomedicine

With the development of electrospinning technology and some breakthroughs in the medical field, the transparent fibrous membrane has been widely used in the biomedical field due to its simple production method and high specific surface area [121,122]. For example, the use of a highly transparent electrospinning fibrous membrane as wound dressing is conducive to doctors’ intuitive inspection of wound conditions and avoids secondary damage caused by frequent opening of the dressing [123]. Compared with ordinary fibers, electrospun polymer fibers have better biocompatibility and can promote cell differentiation [124], so they can be used as a scaffold for cell and tissue culture. An electrospun transparent fibrous membrane is more convenient for observing the morphology and growth of cells compared with that of opaque, so as to avoid pollution caused by unnecessary turnover [125]. Jin et al. [126] obtained an electrospun membrane with a diameter less than 1 μm by electrospinning the mixture of natural protein silk and polyethylene oxide (PEO), which was used as a biomaterial scaffold material for vascular transplantation or wound dressing. As a natural polymer material, cellular cellulose has good biocompatibility and high transparency, and it can be applied for tissue engineering scaffolds or medical implant materials [127,128]. Electrospun nanofibers based on cell cellulose (BC) promoted the growth and differentiation of tissue cells, which had a widespread application as a tissue cell growth scaffold [129] and wound-healing platform [130]. Kim et al. [131] found that BC-based electrospun nanofibers had high transparency, which were employed for use as nanopatches to treat traumatic tympanic membrane perforation. Ma et al. [80] electrospun a PCL/shellac transparent fibrous membrane, which had an excellent drug release performance. The drug release time and amount significantly increased after ethanol treatment, which met the utilization requirements in facial skin care and overnight medication.

After electrospinning and heat treatment, a poly(ethyl 2-cyanoacrylate) (PECA) fibrous membrane with optical transparency and good biocompatibility was prepared [96]. Cells adhered well to the above material for proliferation, which enhanced the metabolic activity and could differentiate in the myotube. These properties of the PECA fibrous membrane enabled it to be used as a protective layer for hip and knee joints, coronary stents, and heart valves, as well as for biomedical devices [132].

### 5.5. Sensors

Transparent sensor refers to a light-transmitting device that measures a specific substance and converts it into an output signal according to a certain rule. The development of a transparent sensor can meet specific needs and develop more extensive applications. At present, the research studies of transparent sensors mainly include chemical sensors that detect and monitor dangerous substances in the gas phase, protecting us from pollution and toxic gases [133]. Self-powered sensors convert wasted micromechanical energy into available electrical energy based on the principles of triboelectricity and piezoelectric [134,135,136]. Optical sensors for chromaticity detection, resistance sensors [137], temperature sensors [98], humidity sensors [138], and pressure sensors, etc. converting external stimuli into electronic signals also had been studied [139].

Duy et al. [133] prepared the elastic polyurethane electrospun nanofibrous multilayer sensor with chemical response, which was flexible and stretchable, by electrospun PU nanofibers and reductive polyethylene oxide (R-GO) by self-assembly (LBL), which was used for gas or steam detection. The R-GO/PU devices with four and five layers have excellent sensitivity and mechanical sustainability up to 50% of the strain under static and cyclic tensile tests. Najarzadekan and Sereshti [76] used PA-6 and 1, 10-phenanthroline (1, 10-Phen) as raw materials to prepare an optical sensor with a nanofibrous scaffold structure by electrospinning and impregnation. It could be used for the colorimetric determination of Fe^2+^ and ascorbic acid. Li et al. [140] prepared a serpentine silver nanofibrous elastomer (SNME) by electrospinning with polyvinyl butyral (PVB) and nano silver as raw materials. By adjusting the content of Ag nanoparticles and chemical deposition time, the photoelectric properties of SNME were optimized, and the films with high visible light transmittance of 81% and low resistance of 9.2 Ω·SQ^−1^ were obtained. After 1000 cycles of repeated stretching, SNME still had a good stretching performance. As a wearable resistance sensor, SNME was used to monitor the movement of finger joints. Qiu et al. [94] obtained highly graphitized phenolic resin electrospun fibers/single-layer graphene (GFG) fibrous membrane by electrospinning, annealing, and solvent evaporation. It had high electrical conductivity and 83% high light transmittance and could be used as a wearable medical sensor for effective skin surface monitoring of biological signals. The fibrous membrane mimicked the mechanical structure of a bird’s nest and significantly enhanced the mechanical properties of the fibrous membrane.

The self-healing TPU nanofibrous film obtained by Choi et al. [141] was opaque. As time went on, the nanofibrous film became irreversibly transparent at room temperature through self-healing-induced interfibrillar fusion, leading to the appearance of a warning sign. It could be applied as a time sensor to detect the change of time and monitor the freshness of food, and it operated through an intrinsic physical response Compared with traditional sensors, electrospun fibrous sensors were more portable and breathable, and they effectively avoided the joint swelling [142] and inflammation [143] caused by wearing for a long time and health detection.

### 5.6. Energy and Optoelectronics

Transparent fibrous film has been widely studied in the field of optoelectronics, such as flexible transparent display screens [144], transparent electrodes [145,146,147,148], transparent light-emitting diodes [110], stretchable transparent heaters [149], etc. Transparent polymer nanofilms reinforced by fibrous can effectively replace indium tin oxide (ITO) as a new electrode material [150]. Wang et al. [151] used electrospun pan as a mask, polyethylene terephthalate (PET) and polydopamine (PDA) as a substrate, and metal Ag as a raw material to prepare metal mesh with super adhesion and mechanical strength. The silver grid (s-Ag) mainly provided high conductivity, while polymer PDA with strong adhesion provided mechanical flexibility. When the composite material was used as the electrode of a solar cell, the power conversion efficiency was 6.4%, and it had superior mechanical stability and high conductivity. Matei et al. [105] electroplated the electrospun PMMA fibrous and prepared the ZnO-coated PMMA fibrous membrane. The fibrous membrane had excellent photocatalytic properties and was used as a transparent electrode of the solar cell. He et al. [152] electrospun metal Cu nanofilms on rigid glass and flexible PET substrates. Except for its conductivity, the fibrous film also showed outstanding toughness, which withstood repeated transparent tape peeling and various bending tests and could be used as a flexible touch screen.

In order to improve the definition of structural metals due to a large scattering cross-section, which affects the application of electrospun metal fibers in touch screens and transparent electrodes, Kim et al. [153] carried out a surface oxidation treatment on electrospun silver nanofibers to form an Ag/Ag_2_O core/shell structure. Ag_2_O had absorption capacity, which eliminated multiple moments in Ag nanowires and inhibited plasma scattering. The transmittance of Ag/Ag_2_O was higher than that of bare Ag nanowires, and the transmittance was not less than 92% in the visible light range.

**Figure 10:**
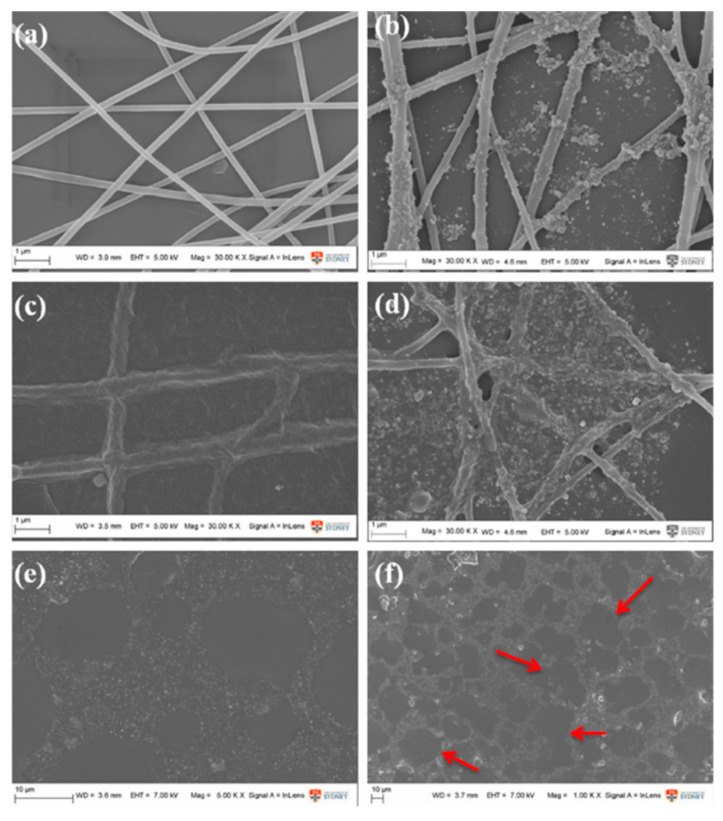
High-resolution SEM images of polyurethane (PU) nanofibrous mats. (**a**) Electrospun for 180 s. PU nanofibrous electrospun for 180 s dipped with (**b**) silver phosphate nanometer particles (AgNPs), (**c**) graphene nano solution (GNSs), (**d**) AgNPs–GNSs (5:1) and (**e**) AgNPs–GNSs (3:1) after melting. (**f**) shows SEM micrograph of (**e**) under low magnification. (Adapted from [155]).

He et al. [154] prepared transparent conductive films with light transmittance of 90% at 550 nm by electrospinning, annealing, and etching using reduced graphene oxide (rGO) and PVA. In general, when the film thickness increased, the conductivity increased, but the transmittance decreased. The method of etching with electrospun PVA fibrous film effectively balanced the relationship between conductivity and transmittance of the rGO conductive nanobelt network. Huang et al. [155] immersed the polyurethane electrospinning nanofibers membrane into the mixture of silver phosphate nanometer particles and graphene nano solution (AgNP–GNS) to construct two-dimensional conductive networks, obtaining self-assembly AgNP–GNS/PU film, which had great potential in the field of transparent flexible electrodes. In this research, the Ag nanoparticle layer had little effect on light transmittance. The wavelength of visible light was comparable to that of light scattering between the nanofiber gaps, which caused light scattering, so through heating the PU nanofibers until melted, the PU nanofibers gap decreased, and the high light transmittance nanofibrous membrane was obtained (as shown in Figure 10). Tian et al. [156] prepared a ZnO–SnO_2_ nanofibrous membrane by electrospinning, and it was used in transparent devices as a high-performance fully transparent photodetector, which had high UV sensitivity, a photoelectric dark current ratio, and a fast response speed. Firstly, the Zn (NO_3_)_2_ and SnCl_2_ were electrospun with the help of polymer polyvinylpyrrolidone (PVP). The transparent fibrous film was calcined to remove the organic components to obtain zinc–tin oxide nanofibers, and the light transmittance was further improved. 

### 5.7. Oil–Water Separation

Electrospinning fibrous membrane materials are widely used in the field of oil–water separation due to their high porosity, high specific surface area, and good water permeability [59,157]. The ideal oil–water separation membrane with super hydrophobic and high lipophilic could achieve high oil–water separation efficiency. Zhang et al. [158] decorated an UV-resist and transparent coating consisting of PDMS and ZnO on a highly stable and self-standing polyimide. The obtained fibrous membrane had the characteristics of excellent UV resistance and 99% oil–water separation efficiency, and the transmittance was as high as 93%. Herein, PDMS was mainly used to enhance the wettability of PI, while increasing the oil–water separation efficiency of PI fibrous membrane. ZnO provided an anti ultraviolet effect and prolonged the service life of the polymer fibrous membrane. Ma et al. [159] also used PDMS to treat PI to obtain a PI fibrous membrane with significantly enhanced oil–water separation efficiency. Different from Zhang’s research, the anti-ultraviolet property was mainly provided by the tannin of plant polyphenols, and tannic acid had an inherent ultraviolet absorption capacity. Therefore, the service life of the modified PI fibrous membrane was prolonged and it had high transparency.

### 5.8. Food Packaging

Food packaging materials are required to be safe and non-toxic. Their water resistance, gas barrier, and mechanical properties should be excellent as well. In general, the electrospun fibers has a large aspect ratio, which establishes a tortuous path to provide a channel for the diffusion of oxygen molecules and performs as a reduced barrier. However, some researchers relied on the establishment of hybrid fibers that blocked the oxygen diffusion path and performed as an excellent gas barrier, which met the requirements of food packaging materials [160,161].

In order to meet the material safety requirements of food packaging, safe and non-toxic natural polymers are usually selected, such as oregano essential oil for its significant antibacterial effect [162]. Lopez produced packaging films of poly(3-hydroxybutyrate-co-3-hydroxyvalerate) (PHBV) with alpha- and gamma-cyclodextrins (α-CD and γ-CDs) containing oregano essential oil (OEO). After annealing, transparent films with enhanced antioxidant and antibacterial properties were obtained, which prolonged the time of food preservation effectively when used in food packaging. Poly (3-hydro-xybutyrate) (PHB) is a non-toxic, harmless, and biocompatible material, which is widely used in food packaging. After annealing, the PHB film was uniform and transparent. The oxygen permeability of the film was significantly reduced, and the water resistance of the film was also significantly improved [90].

A nanofibrous membrane with antibacterial property is also a major development direction of food packaging materials. Food poisoning is usually caused by histamine produced by food-borne bacteria such as Staphylococcus aureus, Proteus and Salmonella paratyphi A. Erbay et al. [163] prepared a submicron PCL nanofibrous membrane filled with nano zeolite and silica (SiO_2_) by electrospinning, and then heat-treated it to obtain a transparent fibrous membrane with significantly improved mechanical properties, which effectively captured histamine produced by Staphylococcus aureus and paratyphoid bacillus.

### 5.9. Anti-Icing Coating

The anti-icing coating is an effective method to prevent icing and facilitate de-icing. The anti-icing coating is not only superhydrophobic and ice repellent [164,165] but also cold resistant in a low-temperature environment [166,167]. After improving the surface roughness, porous electrospun fibers are more easily prepared as a superhydrophobic surface, which is effective for anti-icing coating. Tas et al. [168] injected lubricants matching its refractive index into electrospun PVDF-co-HFP, such as poly (chlorotrifluoroethylene) (PcTFE) or silicone oil, and the light transmittance of the fibrous film was as high as 90%. The results showed that the lubricant provided a smooth surface for the electrospun fibrous film, and the ice adhesion was significantly reduced.

### 5.10. Anti-Corrosion Material

Coatings, corrosion-resistant materials, and corrosion inhibitors are usually the main strategies to solve the problem of steel corrosion [169]. Metal corrosion is caused by the direct contact between metal and water, oxygen, or acid solution, so the effective method of metal corrosion prevention is to isolate the metal itself from contacting with water, oxygen, or acid solution [170,171]. Usually, an anti-corrosion coating with a superhydrophobic surface could effectively reduce the contact range between the metal matrix and corrosion solution [172]. Therefore, some self-healing material due to its specific property could be applied as an anti-corrosion material, which not only has good anti-corrosion effect but also has significantly enhanced durability [173]. An et al. [174] prepared two core–shell structured fibers by coaxial electrospinning. One of them was liquid resin monomer in the core and PAN in the shell, and the other was a liquid-curing agent in the core and PAN in the outer layer. These two kinds of fibers were intertwined and embedded into PDMS or SU-8 to form a transparent flexible self-healing composite, which was covered on the surface of the metal. When the surface was scratched, the liquid curing agent and resin were released from the fibers core, polymerized, and repaired the damaged surface to protect the metal from corrosion.

## 6. Conclusions

On the basis of the present research situation and prospects, a facile electrospinning strategy was implemented to fabricate a fibrous membrane for achieving an enhanced transparency, which had a wide application prospect in optical devices, flexible display screens, light-emitting diodes, filtering, self-cleaning, cell growth scaffolds, wound dressing, and drug release carriers. Investigating the light transmission mechanism of electrospinning fibers, research studies based on the materials selection, controllable microstructure, and topological arrangement were conducted, and several methods were employed to provide effective solutions to obtain homogenous membranes with comparable refractive indexes beneficial for light propagation. However, some restrictions still exist, including the reduced porosity and thickness of the fibrous membrane, which destroys its internal structure and mechanical properties. Therefore, to balance the methods/parameters and thickness of the fibrous membrane with the mechanical properties, the thermal properties and stability of membrane materials still need to be explored by researchers.

## Figures and Tables

**Table 1:** Materials, preparation methods, film thickness, average diameter, and application fields of nanofibrous films with light transmittance above 90%.

Material	Light Transmittance	Thickness	Average Diameter	Preparation Method	Application	Citation
CNWs/epoxy resin	92%	0.43 ± 0.03 mm		Dipping after electrospinning	reinforcing material	[78]
PAN/PMMA	90%	0.1–0.4 mm	550 nm	Dipping after electrospinning	windshields for fast-moving objects	[55]
PA-6/TPU	>90%		163 nm	Casting after electrospinning		[64]
ITO/PVP	95%	75.7 nm	188.0 ± 29.6 nm	Annealing after electrospinning	optoelectronic and sensing applications.	[87]
PAN/PU	>95%			Thermal treatment after electrospinning	temperature monitor	[98]
Cu	90%			Surface modification after electrospinning	transparent heater	[109]
ZnO/ PDMS-PI	93%			Surface modification after electrospinning	oil–water separation membrane	[158]
Ag/Ag_2_O	92%			Surface modification after electrospinning	optoelectronic applications	[153]
Cu/Ni	93%		4.75 μm	Surface modification after electrospinning	transparent heater	[113]
rGO/PVA	90%			Annealing after electrospinning	conductive film	[154]
PDMS/PAN	90%			Direct electrospinning	self-healing anticorrosive material	[174]
AgNW CuNW	90%		70 nm	Electroless deposition after electrospinning	electrical conductivity	[103]
Cu				Electroless deposition after electrospinning	transparent electrode	[104]

## Data Availability

The data presented in this study are available on request from the corresponding author.
